# Supplementary material for: Electromagnetic navigation for femoral osteotomy using high-accuracy X-ray-to-CT registration
Source: Int J Comput Assist Radiol Surg. 2026 Jun 20;21(11):2219–29. doi: 10.1007/s11548-026-03726-9 (PMC13759605; doi:10.1007/s11548-026-03726-9)
Supplement: Supplementary file 1 [file 11548_2026_3726_MOESM1_ESM.pdf]

Supplementary Material: Electromagnetic Navigation for Femoral Osteotomy Using High-Accuracy X-ray-to-CT Registration

Roman Flepp[†1,2*], Arend Nieuwland[†1,3*], Bastian Sigrist[2], Philipp Fürnstahl[2], Lilian Calvet[‡2], Thomas Dreher[‡1,3]

[1]Department of Pediatric Orthopedics and Traumatology, University Children's Hospital Zürich, Switzerland.
[2]Research in Orthopedic Computer Science, University Hospital Balgrist, University of Zurich, Switzerland.
[3]Department of Orthopedic Surgery, University Hospital Balgrist, University of Zurich, Switzerland.
[†]These authors contributed equally.
[‡]These authors jointly supervised this work and share last authorship.

*Corresponding author(s). E-mail(s): roman.flepp@kispi.uzh.ch; arend.nieuwland@balgrist.ch;

1 Error Analysis

This section provides more details about our data as well as give more interpretations regarding the difficulty levels A, B and C.

1.1 Raw Error Data

This section presents the raw residual error measurements for all $N = 18$ osteotomy trials. The data is grouped by the navigation method (EMT, Free-hand, PSI). Table 1 details the rotational deviations decomposed into clinically relevant angles of derotation, varisation and extension as well as the translational deviations (x, y, z). A graphical representation can be found in Fig. 1.

To give a visual example of the reached corrections, you can find a 3D visualization of the 3D segmented bone models after correction compared to the wanted correction for free-hand and EMT in Fig. 2.

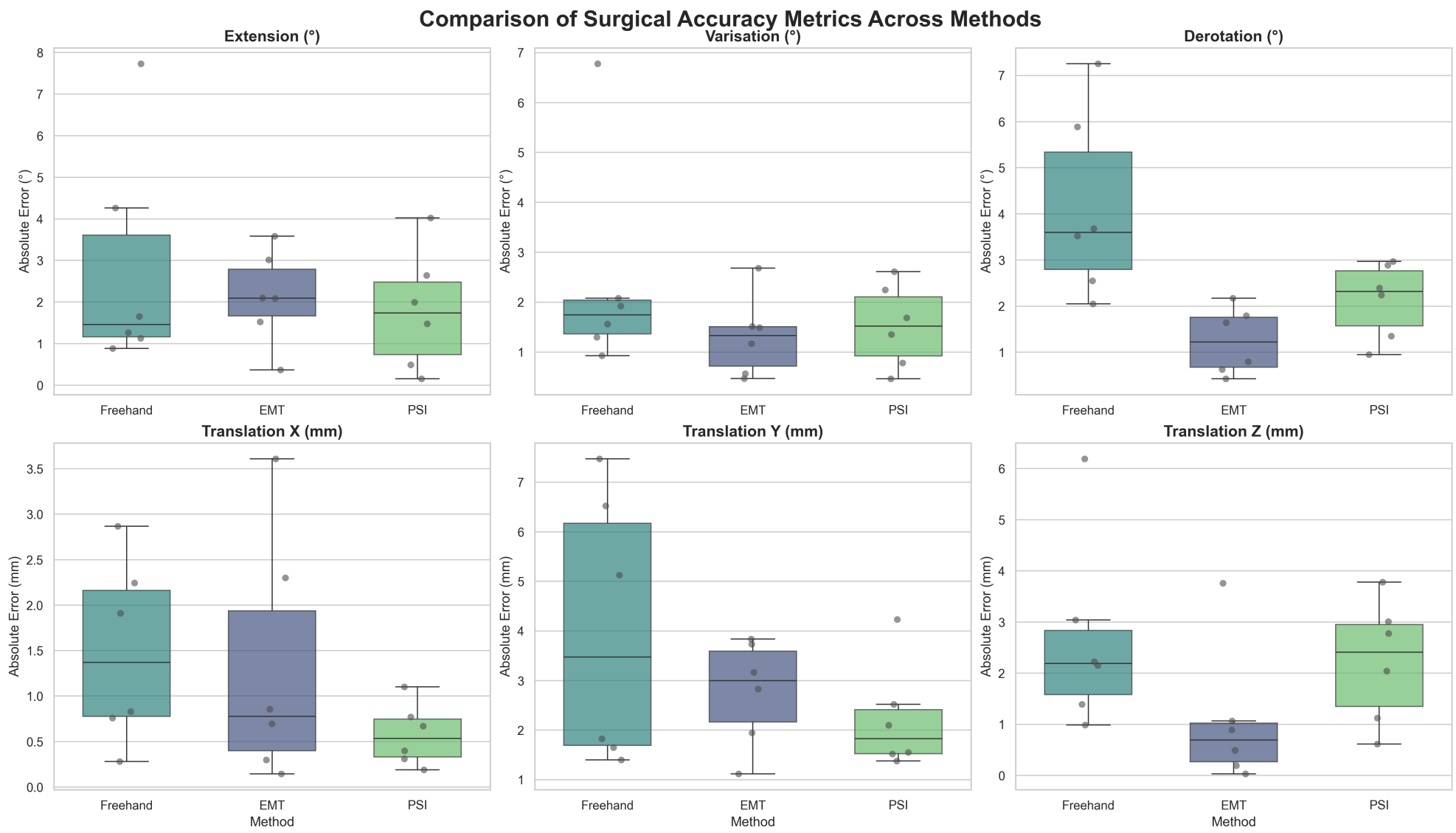

Fig. 1 Plots showing the different error components for the rotational and translational parts.

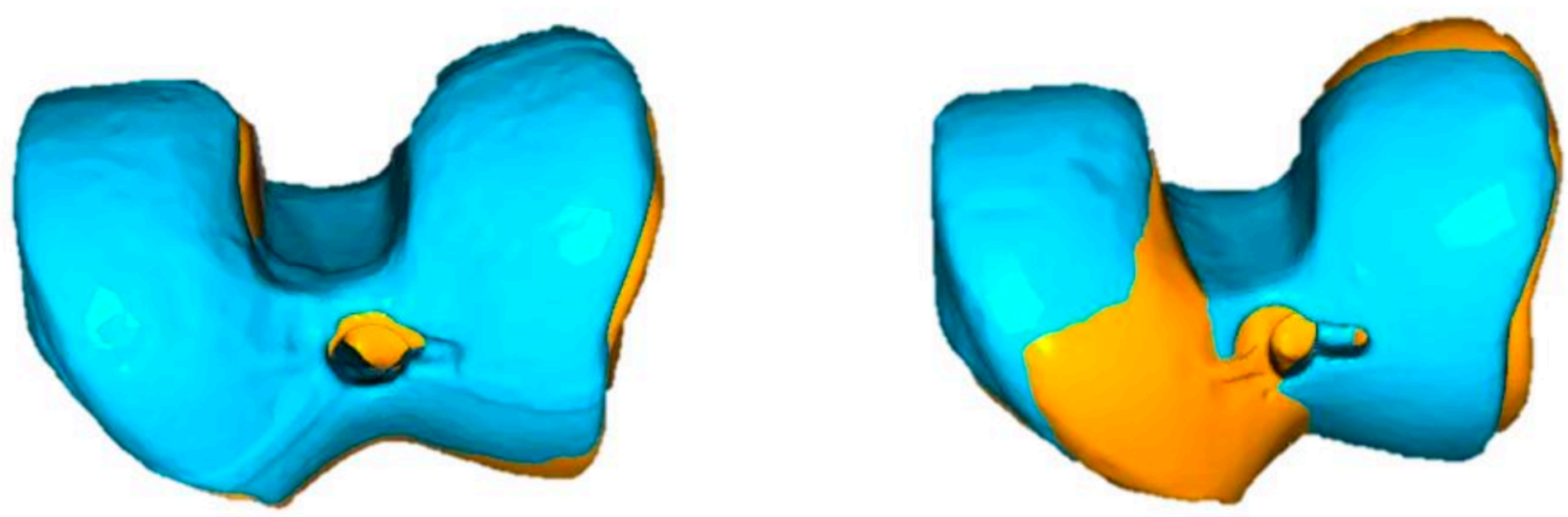

Fig. 2 Yellow is the 3D ground truth of the wanted correction, blue is the achieved correction. On the left is the EMT method and on the right is the free-hand method for the same difficulty level and surgeon.

1.2 Difficulty Levels

Figure 3 illustrates the distribution of angular errors across the three difficulty levels. While the Free-hand technique exhibits increased error and variance as complexity increases (from Level A to C), the EMT-guided trials maintain a consistent accuracy profile comparable to PSI, even in the complex biplanar scenario (Level C).

Method	Surgeon	Level	Rotation (°)			Translation (mm)		
			Ext.	Var.	Derot.	x	y	z
EMT	Surgeon 1	A	-0.37	1.51	-0.80	0.86	1.94	-0.89
	Surgeon 2	A	-3.58	-1.17	0.43	0.30	-3.84	-0.49
	Surgeon 1	B	2.10	2.68	-0.63	-0.70	3.16	-3.76
	Surgeon 2	B	-1.52	0.47	-2.17	0.14	-1.12	1.07
	Surgeon 1	C	-2.08	1.49	-1.64	2.30	-2.83	-0.03
	Surgeon 2	C	-3.02	-0.57	-1.79	-3.61	-3.73	-0.20
Free-hand	Surgeon 1	A	0.88	-1.56	-3.52	-0.76	1.83	2.23
	Surgeon 2	A	-1.65	0.93	-2.05	0.83	-1.40	-2.15
	Surgeon 1	B	-1.13	6.78	2.55	1.91	-1.65	-6.19
	Surgeon 2	B	4.26	2.08	-5.89	2.25	7.47	-3.04
	Surgeon 1	C	-7.73	-1.30	3.68	0.28	-6.52	-0.99
	Surgeon 2	C	-1.27	-1.93	7.26	-2.87	-5.13	1.39
PSI	Surgeon 1	A	0.16	1.68	0.95	0.40	-1.52	1.12
	Surgeon 2	A	2.00	0.47	1.35	-0.31	1.55	2.04
	Surgeon 1	B	0.49	2.61	2.97	1.10	-2.52	-0.61
	Surgeon 2	B	-4.02	2.25	-2.39	0.19	-4.23	-2.78
	Surgeon 1	C	-1.48	-0.78	2.89	0.77	-2.10	3.78
	Surgeon 2	C	2.64	-1.35	2.24	0.67	1.38	3.01

Table 1 Raw rotational and translational error measurements grouped by method. Abbreviations: Ext. (Extension), Var. (Varisation), Derot. (Derotation).

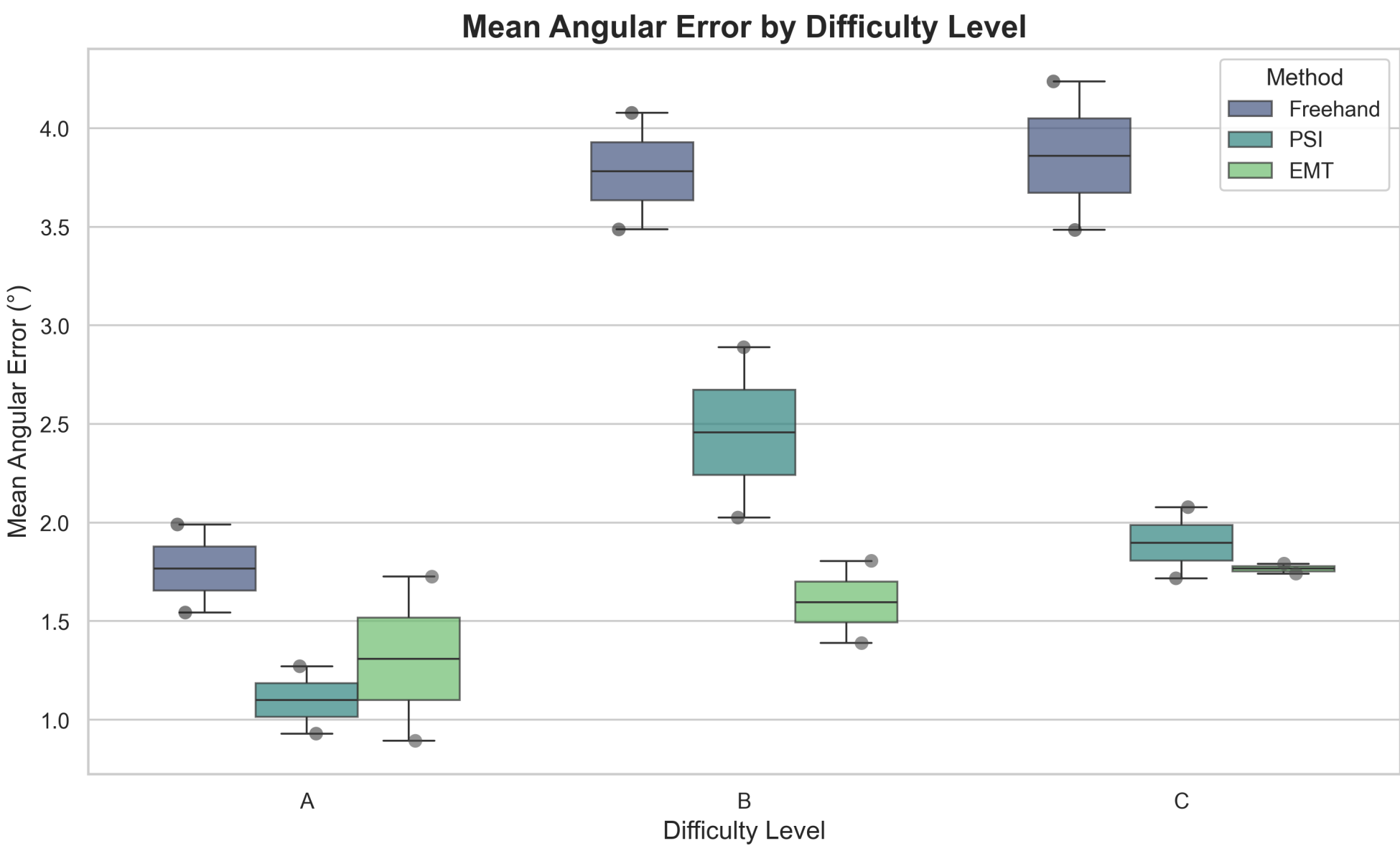

Fig. 3 Boxplots of Mean Angular Error (°) stratified by difficulty level (A: Simple, B: Complex, C: Biplanar) and navigation method. The boxes represent the interquartile range (IQR), the horizontal line indicates the median, and the overlaid points represent the individual osteotomy trials. Note the tight clustering of EMT results across all levels, contrasting with the wider dispersion observed in the Free-hand group at higher complexities.

2 Normality Analysis and Statistical Justification

2.1 Methodology

The data points of our feasibility study were analyzed as a complete block design with six matched blocks (2 surgeons $\times$ 3 difficulty levels). Although Shapiro-Wilk tests indicated no significant deviation from normality (all $p > 0.05$), non-parametric methods were chosen due to the limited statistical power inherent in the small sample size of ($n = 6$) trials per method. We pre-planned and assessed the superiority of EMT vs. Free-hand and the equivalence of PSI vs. EMT.

Superiority was evaluated using a one-sided Wilcoxon signed-rank test ($\alpha = 0.05$). Equivalence was assessed using a non-parametric two one-sided tests (TOST) procedure with symmetric bounds of $\delta = 2.0°$ and $\delta = 2.0$ mm. Results report differences with 95% confidence intervals (CI) for superiority and 90% CI for equivalence. Descriptive statistics denote pooled mean $\pm$ standard deviation (SD).

2.2 Equivalence Bounds

The equivalence margins of $\pm 2°$ and ± 2 mm were constrained from three independent directions, such that tightening the margin further would violate at least one of them.

Upper bound: clinical meaningfulness. The established clinical threshold for residual malalignment associated with osteoarthritis progression and the need for revision surgery in femoral osteotomies is 5° [1], and most clinical guidelines consider deviations greater than 5° from neutral position as the threshold warranting surgical concern [2, 3]. For translational accuracy, offsets at the osteotomy site propagate directly into mechanical axis deviation (MAD) of the lower limb, for which a post-operative tolerance of $\sim$10 mm is widely accepted [3]. We selected $\pm 2°$ and ± 2 mm, corresponding to $\leq 40\%$ of these clinical tolerances.

Lower bound: system measurement precision. The margin must also exceed the combined measurement uncertainty of the navigation error chain, otherwise TOST would be noise-limited and an equivalence verdict would be uninformative. As reported in Section 3.1, a root-sum-of-squares combination of the dominant error sources (see response 4.) yields an end-to-end translational uncertainty of ≈ 0.89 mm. A ± 2 mm / $\pm 2°$ margin sits comfortably above this floor, ensuring that a positive equivalence result reflects genuine agreement between methods rather than limits of the measurement system.

Reference-method variability. Finally, the margin should not fall below the intrinsic variability of the active comparator (PSI), since a tighter δ could not be satisfied even when benchmarking PSI against itself. Neopoulos et al. [4] reported mean absolute errors of $1.1° \pm 1.0°$ (varus) and $2.4° \pm 1.9°$ (derotation) in distal femoral osteotomy with combined coronal–rotational correction, while Fucentese et al. [5] reported 3D translational errors of 0.1 ± 1.3 mm in the coronal plane and $0.1° \pm 0.6°$ mm in the sagittal and transversal planes for PSI-guided osteotomies. These place state-of-the-art PSI performance at or near the chosen bounds, confirming that $\pm 2°$ and ± 2 mm represent a tight margin achievable given the reference method's own reproducibility.

Summary. Taken together, the $\pm 2°$ / ± 2 mm margin is the narrowest bound simultaneously satisfying: (i) clinical meaningfulness ($\leq 50\%$ of failure thresholds), (ii) system measurement precision (> 0.89 mm uncertainty), and (iii) reference-method capability (at or above reported PSI variability). We have added this context and the corresponding references to Section 3.3 of the revised manuscript, with the full justification provided in the supplementary material.

2.3 Normality Tests (Shapiro-Wilk)

All Shapiro-Wilk tests, see Table 2, yielded p-values greater than 0.05, indicating no significant deviation from normality for the paired differences.

Metric	Contrast	W-statistic	p-value	Result
**Varisation**	EMT vs Free-hand	0.8379	0.1253	Normal
	PSI vs EMT	0.9142	0.4648	Normal
**Derotation**	EMT vs Free-hand	0.8680	0.2185	Normal
	PSI vs EMT	0.8834	0.2851	Normal
**Extension**	EMT vs Free-hand	0.8838	0.2871	Normal
	PSI vs EMT	0.8214	0.0908	Normal

Table 2 Shapiro-Wilk normality test results for paired differences. W-statistic denotes the test statistic.

2.4 Statistical Reasoning

Despite the Shapiro-Wilk tests failing to reject the null hypothesis of normality ($p > 0.05$ for all extensions), we prioritized non-parametric Wilcoxon Signed-Rank Test to assess superiority in our statistical analysis. The following reasons lead us to this approach:

1. **Low Statistical Power**: With a small sample size of $n = 6$, normality tests have low power. This means they are prone to failing to detect non-normality when it actually exists. A non-significant result in this context is often a reflection of sample size rather than true normality.
2. **Robustness**: Non-parametric tests make fewer assumptions about the underlying distribution. Given the potential for outliers in surgical navigation data and the small sample size, the Wilcoxon Signed-Rank test provides a more robust estimate of the treatment effect than the paired t-test, which is sensitive to outliers.
3. **Conservative Approach**: Choosing a non-parametric test is a conservative strategy that avoids the risk of relying on parametric assumptions that cannot be reliably verified with $n = 6$.

Thus, the choice of the Wilcoxon Signed-Rank test is methodologically sound and consistent with best practices for small-sample surgical studies.

References

[1] Sharma, L., Song, J., Felson, D.T., Cahue, S., Shamiyeh, E., Dunlop, D.D.: The role of knee alignment in disease progression and functional decline in knee osteoarthritis. JAMA **286**(2), 188–195 (2001) https://doi.org/10.1001/jama.286.2.188

[2] O'Malley, M.P., Pareek, A., Reardon, P.J., Stuart, M.J., Krych, A.J.: Distal femoral osteotomy: Lateral opening wedge technique. Arthroscopy Techniques **5**(4), 725–730 (2016) https://doi.org/10.1016/j.eats.2016.02.037

[3] Paley, D.: Principles of Deformity Correction. Springer, Berlin, Heidelberg (2002). https://doi.org/10.1007/978-3-642-59373-4

[4] Neopoulos, G., Jud, L., Vlachopoulos, L., Fucentese, S.F.: Combined correction of coronal and rotational deformities of the femur with distal femoral osteotomy using patient-specific instrumentation. The American Journal of Sports Medicine **53**(4), 848–854 (2025) https://doi.org/10.1177/03635465251314868

[5] Fucentese, S.F., Meier, P., Jud, L., Köchli, G.-L., Aichmair, A., Vlachopoulos, L., Fürnstahl, P.: Accuracy of 3d-planned patient specific instrumentation in high tibial open wedge valgisation osteotomy. Journal of Experimental Orthopaedics **7**(1), 7 (2020) https://doi.org/10.1186/s40634-020-00224-y
